# Prediction of Johne’s disease state based on quantification of T cell markers and their interaction with macrophages in the bovine intestine

**DOI:** 10.1186/s13567-021-00925-x

**Published:** 2021-04-13

**Authors:** Caitlin J. Jenvey, Adrienne L. Shircliff, Elsa Obando Marrero, Judith R. Stabel

**Affiliations:** 1 USDA-Agricultural Research Service (ARS), National Animal Disease Center, Ames, IA USA; 2 Department of Animal, Plant and Soil Sciences, AgriBio Centre for AgriBioscience, La Trobe University, Bundoora, VIC Australia

**Keywords:** Bovine, CXCR3, Macrophage, *Mycobacterium avium* subsp. *paratuberculosis*, T cell, Johne’s disease

## Abstract

Cell-mediated immune responses to *Mycobacterium avium* subsp. *paratuberculosis* (MAP) are regulated by various types of T lymphocytes. The aim of this study was to quantitate T cell subsets in the mid-ileum of cows naturally infected with MAP to identify differences during different stages of infection, and to determine whether these subsets could be used as predictors of disease state. Immunofluorescent labeling of T cell subsets and macrophages was performed on frozen mid-ileal tissue sections archived from naturally infected dairy cows in either subclinical or clinical disease status, and noninfected control cows. Comprehensive IF staining for CD4, CD8α, TcR1-N24 (gamma delta), FoxP3, CXCR3 and CCR9 served to define T cell subsets and was correlated with macrophages present. Clinically affected cows demonstrated significantly higher numbers of CXCR3^+^ (Th1-type) and CCR9^+^ (total small intestinal lymphocytes) cells at the site of infection compared to the subclinical cows and noninfected controls. Further, predictive modeling indicated a significant interaction between CXCR3^+^ and AM3K^+^ (macrophages) cells, suggesting that progression to clinical disease state aligns with increased numbers of these cell types at the site of infection. The ability to predict disease state with this model was improved from previous modeling using immunofluorescent macrophage data. Predictive modelling indicated an interaction between CXCR3^+^ and AM3K^+^ cells, which could more sensitively detect subclinical cows compared to clinical cows. It may be possible to use this knowledge to improve and develop an assay to detect subclinically infected animals with more confidence during the early stages of the disease.

**Supplementary Information:**

The online version contains supplementary material available at 10.1186/s13567-021-00925-x.

## Introduction

Johne’s disease (JD) is a chronic gastroenteritis of ruminants caused by *Mycobacterium avium* subspecies *paratuberculosis* (MAP). The initial host response to infection involves a strong cell-mediated immune response, which helps to sustain animals in a state of subclinical disease. At some point, this cell-mediated immune response switches to a strong humoral immune response, usually during advanced clinical disease. Cell-mediated immune responses to MAP are regulated by various types of T lymphocytes. CD4^+^ T cells tend to predominate at the site of initial MAP infection and during the early stages of the disease, shifting towards stronger CD8^+^ T cell responses in the later stages of subclinical disease [1]. Secretion of cytokines by CD4^+^ T cells directs the immune response, differentiating into various subtypes including Th1, Th2 and Th17 which guide cell-mediated immune function and humoral immune responses. Gamma/delta (γδ) T cells make up a small proportion of the total T lymphocyte population yet appear to play a key role in the regulation of inflammation and granuloma development during the initial phase of the adaptive immune response to JD [2]. Specifically, the number of CD4^+^ and γδ T cells decrease and increase, respectively, from subclinical to clinical disease states, while CD8^+^ T cells tend to be similar between disease states [3]. Regulatory T cells (Tregs) exert immunomodulatory effects via IL-10 and TGF-β to balance the localized inflammation within the target tissue. Populations of CD4^+^CD8^+^FoxP3^+^ Tregs have been shown to develop in response to low level antigenic stimulation with MAP [4], and the number of FoxP3^+^ cells increase with increased severity of intestinal lesions [5]. Despite these previous studies, there is little information on how the dynamics of T cell subsets at the site of infection in JD influences disease state. In addition, there has been no quantification of CXCR3^+^ and CCR9^+^ T cells in cows naturally infected with MAP, which are preferentially expressed by Th1-type [6] and small intestinal T cells [7], respectively. The aim of this study was to quantify T cell subsets in the mid-ileum of cows naturally infected with MAP to identify differences during different stages of infection, and to determine whether these subsets could be used as predictors of disease state. The identification of T cells that strongly influence disease state may be a useful tool to focus research towards improving the diagnosis of Johne’s disease.

## Materials and methods

### Animals

Samples of mid-ileal tissue were collected at necropsy from a total of 20 Holstein dairy cows naturally infected with MAP, and 8 noninfected control cows. Holstein dairy cows ranged in age from 4 to 9 years in this study and were placed in three groups consisting of 8 non-infected healthy cows, 10 cows naturally infected with MAP but asymptomatic (i.e. subclinical), and 10 cows with the clinical form of the disease. Prior to necropsy, infection was monitored bacteriologically for fecal shedding of MAP using fecal culture and PCR as previously described [8], as well as serologic tests, such as Herdchek ELISA for serum antibodies (IDEXX, Westbrook, ME, USA) and a modified MAP-specific IFN-γ assay measured in the plasma (Bovigam, Thermo Fisher Scientific, Carlsbad, CA, USA) [9]. In addition to fecal shedding and serologic analysis, clinical cows also demonstrated physical signs of disease, including weight loss and watery diarrhea, which provided further evidence for stratification of cows into groups. Animals categorized as clinical demonstrated serum ELISA antibody titers averaging 2.45 S/P ratio and fecal shedding average 2369 CFU of MAP/g feces. Cows in the subclinical group were ELISA-negative and averaged less than 17 CFU of MAP/g feces. Infected animals in both the subclinical and clinical stages of infection had equivalent positive antigen-specific IFN-γ results (Abs_450nm_MPS − Abs_450nm_NS = 0.20). All animal related procedures, such as euthanasia and necropsy, were approved by the IACUC (National Animal Disease Center, Ames, Iowa, USA).

### Tissue collection and snap-freezing protocol

At necropsy, the entire section of ileum extending from the ileo-cecal valve through the distal flange was excised and then cut equally into proximal, mid- and distal sections. Tissues were rinsed with 0.15 M PBS and cut into multiple cross-sections for the culture of MAP and PCR to assess bacterial burden. Cross-sections immediately adjacent were processed for histopathology and IF labeling. A dry ice bath was prepared by combining 95% ethanol with dry ice and mixed until a slurry consistency was achieved. Isopentane (Sigma-Aldrich, St. Louis, MO, USA) was added to a tin cup and the cup was placed into the dry ice bath. The mid-ileal intestinal samples were washed with PBS, pH 7.4, and a cross-section was positioned luminal side down on a section of liver covered with Tissue-Tek optimum cutting temperature (OCT) compound (Sakura Finetek, Torrance, CA, USA) to protect the villi during the freezing process and to ascertain tissue orientation post-freezing. The intestine-liver sample was wrapped in foil and placed in the isopentane for at least 5 min. The snap-frozen sample was transferred to dry ice for transport to storage at −80 °C, where it remained until tissue sectioning could be performed.

### Tissue sections

The mid-ileal intestinal samples were removed from −80 °C and placed in a cryostat at −20 °C for at least 30 min prior to sectioning. Tissue samples were embedded in OCT, cut in 6 μm sections and adhered to ColorFrost Plus microscope slides (Thermo Fisher Scientific). Tissue sections were allowed to air-dry overnight at room temperature before fixing for 5 min at −20 °C. Tissue sections were stored at −80 °C until histochemistry and IF staining could be performed.

### Primary antibodies

Primary antibodies used to identify different T cell cohorts were as per Table 1. Th1-type immune responses were represented by the following markers: CCR9, a chemokine receptor expressed by small intestinal T cells; CD4, T helper cells; CD8, cytotoxic T cells; and CXCR3, a chemokine receptor that is highly expressed on effector T cells (CD4^+^ and CD8^+^ T cells). Th2-type immune responses were represented by FoxP3, a protein expressed by natural and adaptive/induced Tregs. Th1/Th17 immune responses were represented by TcR1, a protein complex (receptor) found on the surface of γδ T cells. Macrophages were identified using an antibody to a macrophage surface antigen, AM3K.

**Table 1:** **T cell primary antibodies**

Primary	Host	Isotype	Dilution	Manufacturer
CCR9	Rabbit	IgG	1:400	Novus Biologicals, Littleton, CO, USA
CD4	Mouse	IgG2a	1:100	VMRD, Pullman, WA, USA
CD8α	Mouse	IgG1	1:100	VMRD, Pullman, WA, USA
CXCR3	Rabbit	IgG	1:400	GeneTex Inc., Irvine, CA, USA
FoxP3	Mouse	IgG1	1:100	Washington State Monoclonal Antibody Center, Pullman, WA, USA
TcR1-N24	Mouse	IgG2b	1:100	VMRD, Pullman, WA, USA

### Antibody biotinylation

Primary antibodies were biotinylated using an EZ-Link Sulfo-NHS-LC-Biotin and the procedure outlined by Thermo Fisher Scientific. Briefly, 2 mg of biotin was diluted in ultrapure water and diluted 1:25. An appropriate volume of 1:25 biotin solution was added to 100 µL of primary antibody and allowed to incubate at room temperature for 60 min. The biotinylated primary antibodies were added to a 0.5 mL desalting column and centrifuged at 1500 × *g* for 2 min to purify the biotinylated antibody.

### Immunofluorescence protocol

Tissue sections were removed from −80 °C and allowed to equilibrate to room temperature for 10–20 min. A liquid blocker “Pap” pen was used to draw a hydrophobic barrier around the tissue and allowed to dry. Following tissue rehydration, 3,3-diaminobenzidine (DAB, Vector Laboratories, Burlingame, CA, USA) was added for 10 min to quench eosinophil autofluorescence and washed 3 times for 5 min each. A protein, serum-free blocking solution (Agilent Dako, Santa Clara, CA, USA) was added for 30 min to reduce non-specific labeling. The slides were not washed in-between serum blocking and endogenous biotin blocking/primary antibody incubation. For protocols that included biotinylated primary antibodies, slides were blocked for endogenous biotin prior to the incubation of the first biotinylated primary antibody, and in-between biotinylated primary antibodies. Streptavidin and biotin (Thermo Fisher Scientific) were diluted to concentrations of 0.1 mg/mL and 0.05 mg/mL, respectively. Streptavidin was incubated first for 15 min, followed by a washing step. Slides were washed alternately with 0.05M Tris buffer, followed by 0.05M Tris buffer with 0.02% Tween-20 and 0.09% sodium chloride, 3 times for 3 min each. Following washing, biotin was incubated for 15 min, followed by a washing step. The primary and secondary antibodies, their dilutions, incubation times, and manufacturer sources are presented in Tables 1 and 2, respectively. All secondary antibodies were sourced from Thermo Fisher Scientific. The slides were washed after primary and secondary antibody incubation using the method described above. Incubation with a 1:6000 dilution of 4′,6-diamidino-2-phenylindole, dihydrochloride (DAPI) for 10 min was used to counterstain the nuclei. The slides were mounted in ProLong Gold Antifade Mountant (Thermo Fisher Scientific) and Richard-Allen Scientific “Slip-Rite” Cover Glass #1.5 (Thermo Fisher Scientific). The mounting medium was allowed to cure for at least 30 min at room temperature before imaging.

**Table 2:** **T cell secondary antibodies**

Secondary	Reactivity	Host	Isotype	Dilution
Alexa Fluor 488	Mouse	Goat	IgG (H+L)	1:500
Alexa Fluor 488	Mouse	Goat	IgG2a	1:500
Alexa Fluor 488	Mouse	Goat	IgG2b	1:500
Alexa Fluor 594	Rabbit	Goat	IgG (H+L)	1:500
Alexa Fluor 594	Mouse	Goat	IgG (H+L)	1:500
Alexa Fluor 647	Mouse	Goat	IgG (H+L)	1:500

### Confocal imaging

The tissue sections were examined with an A1 Resonance Plus inverted microscope (Nikon, Melville, NY, USA) equipped with a four-laser Gallium-Arsenide-Phosphide/normal Photomultiplier Tube detector unit (DU4) (GaAsP: 488 and 561; PMT: 405 and 640), Galvano resonant scanner and NIS Elements Advanced Research software (version 4.50.00). Images were acquired by sequential scanning to avoid fluorescence cross-over using a 405/488/561/640 dichroic mirror. All slides were imaged using the following bandpass filters: 405 solid-state diode laser and 450/50 nm bandpass filter, 488 nm solid-state diode laser and 525/50 bandpass filter, 561 nm solid-state diode laser and 600/50 bandpass filter, and 640 solid-state diode laser and 685/70 nm bandpass filter. Images were captured using a 60× Plan Apo lambda objective (1024 × 1024 pixels), numerical aperture 0.75, pinhole 1.2 AU, and exposure 6.2 s per pixel dwell. Detector sensitivity (gain) and laser power settings were kept the same for all collected images to allow comparisons between markers and cows. A total of 10 images were collected per cow to perform statistical analysis.

### Determination of T cell number by surface area labeling

Upon collection of each image, spectral profiles for each primary antibody were determined based upon slides containing only a single primary and secondary antibody. Spectral profiles were used to subtract true IF labeling from background. Thresholding was then performed to create a binary layer for each laser channel on which quantitative analysis of IF labeling could be performed. Thresholds were applied to lower and upper intensity limits to reduce the contribution of non-specific immunofluorescent (IF) labeling to binary layer calculations. Additionally, binary layer contours were smoothed and cleaned to remove small objects and reconstruct morphology. To determine the numbers of T cells, the surface area (μm^2^) for each fluorescent marker was measured and was used as an indicator of numbers of cells e.g. the greater the μm^2^, the greater the number of cells. A macro was developed (Nikon Instruments Support Team, Minneapolis, MN, USA) to automatically measure the surface area of each immunofluorescent antibody in all collected images, based upon the pre-determined spectral profiles and thresholding settings. All data was collated in Microsoft Excel prior to statistical analysis.

### Statistical analysis

All statistical analyses were performed using R version 3.6.1 [10]. Frequency histograms (“graphics” package) determined that the data was non-normally distributed with a positive skew. Distributions for T cell markers best fit a Gamma (TcR1-N24, CD4, CD8, and FoxP3), a log normal (CCR9) and a Poisson inverse gaussian distribution (CXCR3). Generalized linear models (GLM) were used to assess differences between disease states (noninfected, subclinical, clinical) for each T cell marker using the package and function “gamlss” [11]. Prior to performing GLMs, a value of 1 was added to all points of each variable to account for zeros within the dataset. Each model was individually assessed using assumptions of linearity, independence and homoscedasticity. Marginal means and standard errors were extracted for each model and back transformed using model coefficients adjusted for Jensen’s inequality using the following formula: $e\left(logx+0.5 \times {\sigma }^{2}\right)$.

Linear regression models were used to assess the predicted probability of disease state (subclinical vs noninfected control; clinical vs subclinical) using the continuous predictor variables of T cell marker and/or total macrophages (AM3K^+^). Models focused on the interaction between these markers, using the package and function “gamlss”. Models were compared using a backwards, stepwise method based upon the Akaike information criterion for finite sample size (“AICc” in package “MuMIn” [12]). Model parameters included a binomial distribution, logit link function and maximum likelihood estimation method. Correlation of predictor variables was checked using the package “DescTools” and the function “SpearmanRho” [13]. Comparisons with *p*-values less than 0.05 were considered to be statistically significant. The predictive probability (PP) (± 95% confidence intervals) of “disease state” was calculated using the “effects” package [14] and the function “predictorEffect” [15] and plotted with the package “ggplot2” [16] in R.

## Results

### Differences in T cell markers between disease states

In a previous study, our group was able to predict clinical and subclinical disease states by the number of macrophages localized within the mid-ileum of naturally infected cows using generalized logistic regression [17]. In the present study, generalized linear models were used to assess T cell subsets and determine whether specific T cell subsets or T cell markers corresponded to a specific disease state of the cow (noninfected control, subclinically infected or clinically infected). Of the markers investigated, all were significantly dependent on disease state (*p* < 0.0001) (Table 3). Estimated marginal means (EMM) for numbers of CCR9^+^ (small intestinal T cells; 140.15 ± 1.31 μm^2^) and CXCR3^+^ (Th1-type T cells; 1538.85 ± 13.66 μm^2^) cells were significantly higher in the mid-ileum of clinically infected cows, compared to both subclinically infected and noninfected control cows. EMM for numbers of CD8^+^ (842.23 ± 1.19 μm^2^), and gamma delta T cells (TcR1-N24^+^; 1222.86 ± 1.13 μm^2^) were significantly higher in subclinically infected cows, compared to both clinically infected and noninfected control cows. Additionally, EMM for numbers of FoxP3^+^ cells were significantly higher in noninfected control cows (1111.78 ± 1.11 μm^2^), compared to both subclinically and clinically infected cows. While there was no observable statistical difference between the number of CD4^+^ cells in the mid-ileum of either noninfected control or subclinically infected cows (585.79 ± 1.23 μm^2^ and 585.21 ± 1.21 μm^2^, respectively), both groups had significantly higher numbers of CD4^+^ T cells compared to clinically infected cows (Table 3).

**Table 3:** **Estimated marginal means of T cell marker surface area (μm**^**2**^**) in cows**

	Status		
	Control	Subclinical	Clinical
CCR9	23.23 ± 1.34	20.98 ± 1.31	140.15 ± 1.31
Pr(> t)	1.89E−44	2.27E−49	4.12E−89
CD4	585.79 ± 1.23	585.21 ± 1.21	424.80 ± 1.21
Pr(> t)	6.39E−119	2.05E−129	3.11E−125
CD8α	810.48 ± 1.21	842.23 ± 1.19	514.45 ± 1.19
Pr(> t)	1.71E−127	5.36E−140	8.33E−132
CXCR3	116.29 ± 14.81	177.98 ± 14.21	1538.85 ± 13.66
Pr(> t)	4.99E−16	3.42E−27	3.77E−76
FoxP3	1111.78 ± 1.11	711.20 ± 1.10	520.61 ± 1.10
Pr(> t)	6.61E−177	4.21E−182	1.45E−176
TcR1-N24	1194.59 ± 1.15	1222.86 ± 1.13	506.25 ± 1.13
Pr(> t)	2.10E−150	3.91E−163	1.87E−148

Estimated marginal means (+ 1 ± SEM) were generated by generalized linear mixed modeling using the following formula: Tcell ~ Status for subclinically (*n* = 10) and clinically (*n* = 10) cows infected with MAP, as compared to noninfected control cows (*n* = 8).

SEM, standard error of the mean; Pr(> t), *p*-value for t statistic.

### Models to predict disease state

In an effort to understand the roles that specific T cell subsets play during different stages of MAP infection, and if these markers could be used to predict disease state, multinomial linear mixed models and binomial regression models, which used either individual markers, or interactions between markers as predictors, were assessed. The ability of these models to predict disease state were also assessed when including an interaction with the macrophage marker, AM3K.

#### Model to predict clinical disease state

**Figure 1:**
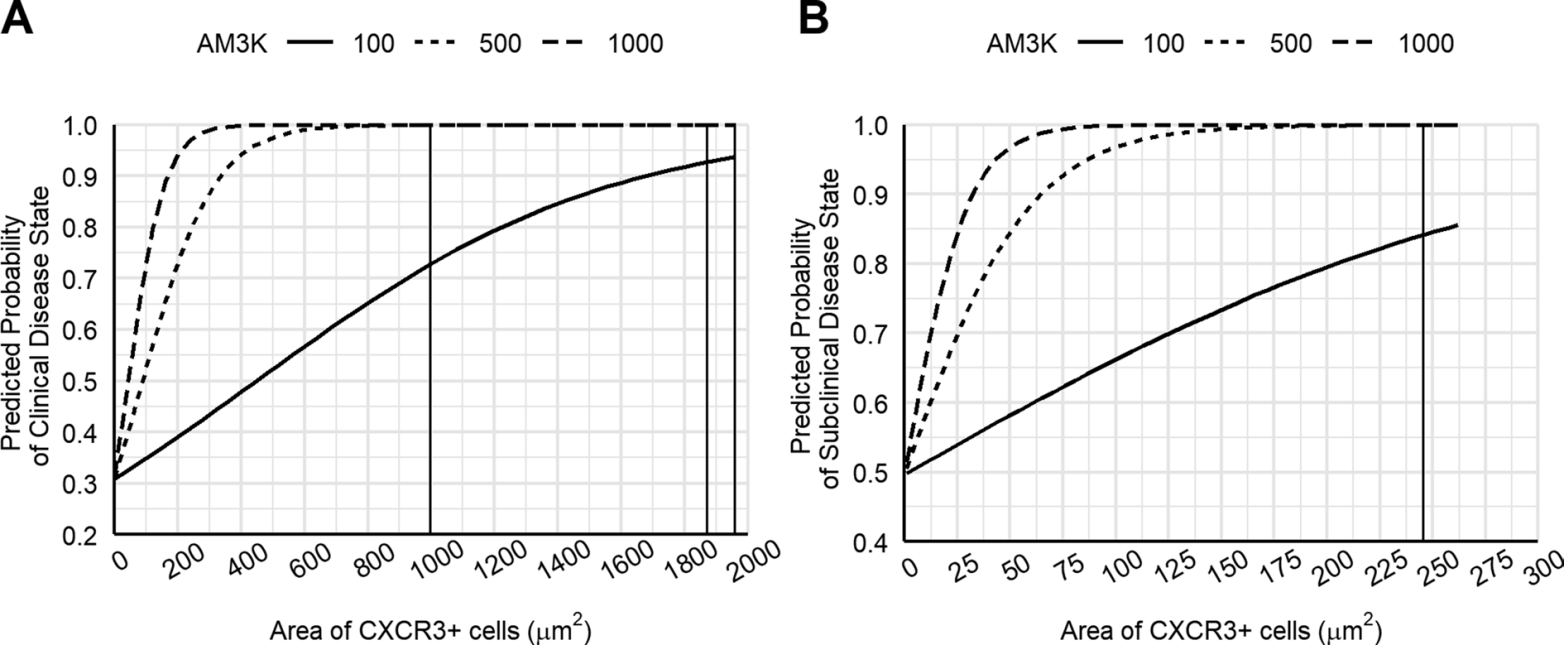
**Binomial linear regression model using CXCR3**^**+**^** and AM3K**^**+**^** cells to predict disease state in dairy cows.** The surface area (μm^2^) of CXCR3^+^ (T cell), and AM3K^+^ (macrophage) cells in the mid ileum was used to predict the probability of clinical (**A**) and subclinical (**B**) disease. Lines represent different numbers of AM3K^+^ cells; 100 μm^2^ (solid line), 500 μm^2^ (dashed line), 1000 μm^2^ (dotted line). Vertical lines indicate the optimal number of CXCR3^+^ cells for each comparison of AM3K^+^ cells.

Modelling indicated that clinical disease state was significantly associated with the interaction between CXCR3 and AM3K, as compared to the PP of subclinical disease state (AICc = 205.37; Pr (> z) = 0.0004). The PP of clinical disease state increased with an increase in CXCR3^+^ and AM3K^+^ cells, achieving 100% (99.9–100%) at 1000 μm^2^ of CXCR3^+^ and 1000 μm^2^ of AM3K^+^ cells, compared to 99.9% (54.9–99%) at 1880 μm^2^ of CXCR3^+^ and 500 μm^2^ of AM3K^+^ cells, and 93.7% (70.7–98.9%) at 1960 μm^2^ of CXCR3^+^ and 100 μm^2^ of AM3K^+^ cells (Figure 1A).

**Figure 2:**
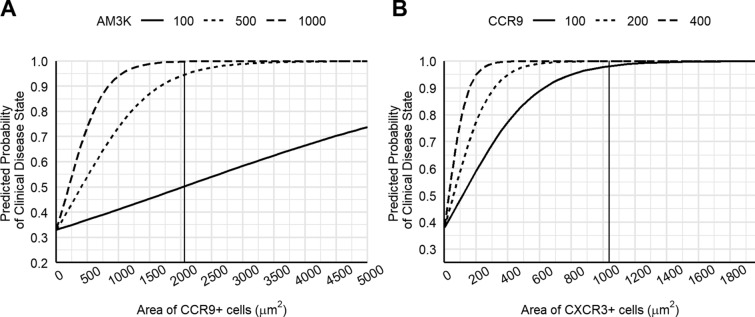
**Binomial linear regression model using CCR9**^**+**^**, CXCR3**^**+**^**, and AM3K**^**+**^** cells to clinical disease in dairy cows.** The surface area (μm^2^) of **A** CCR9^+^ (T cell) and AM3K^+^ (macrophage) cells, and **B** CXCR3^+^ (T cell) and AM3K^+^ cells in the mid ileum was used to predict the probability of clinical disease. Lines represent different numbers of AM3K^+^ cells; 100 μm^2^ (solid line), 500 μm^2^ (dashed line), 1000 μm^2^ (dotted line). Vertical lines indicate the optimal number of CCR9^+^ (**A**) and CXCR3^+^ (**B**) cells for each comparison of AM3K^+^ cells.

In comparison, the interaction between CCR9^+^ and AM3K^+^ cells (AICc = 234.75; Pr (> z) = 0.0001) achieved 99.9% PP with as low as 2200 μm^2^ of CCR9^+^ cells and 1000 μm^2^ of AM3K^+^ cells (98.4–99.9%) (Figure 2A). There was also a significant interaction between CXCR3^+^ and CCR9^+^ cells (AICc = 237.13; Pr (> z) = 0.0004) which achieved 100% PP (99.9–100.0%) at 1040 μm^2^ of CXCR3^+^ cells and 400 μm^2^ of CCR9^+^ cells (Figure 2B).

#### Model to predict subclinical disease state

Modelling indicated that subclinical disease state was significantly associated with the interaction between CXCR3 and AM3K, as compared to the PP of noninfected control state (AICc = 240.98; Pr (> z) = 0.048). The PP of subclinical disease state improved with increased numbers of CXCR3^+^ and AM3K^+^ cells, achieving 100% (56.2–100%) at 246 μm^2^ of CXCR3^+^ cells and 1000 μm^2^ of AM3K^+^ cells, compared to 99.9% (54.9–99%) at 500 μm^2^ of AM3K^+^ cells and 84.2% (53.2–96.1%) at 100 μm^2^ of AM3K^+^ cells (Figure 1B).

## Discussion

Upon infection with MAP, infected animals enter into an often-protracted subclinical stage of infection, which eventually may shift into clinical disease. The mechanism(s) that direct the switch from subclinical to clinical disease is not yet well understood, thus research to gain a better understanding of host immunity to MAP infection is an important step towards effective control. Previous research indicates that there are cellular markers in both the blood and tissue that align with stage of disease. Recent work indicates increased numbers of CD4^+^, CD8^+^ and γδ TCR^+^ T cells in total PBMCs of subclinical cows compared to clinical cows, while stimulation of PBMCs decreases the number of CD4^+^ T cells for all stages of the disease [18]. In intestinal tissue, clinical cows demonstrate significantly higher numbers of total macrophages compared to both subclinical and control cows [19], with a predominant Th2-type macrophage population in clinical cows based on colocalization of cytokines such as IFN-γ, IL-1β, IL-12, uNOS and TNF-α with macrophages [20]. We previously identified that the number of macrophages in the target tissue was an important predictor of Johne’s disease state. However, host immune responses to Johne’s disease appears to be a complex interaction between the many facets of the immune response, therefore, we attempted to identify whether interactions between the T cell and macrophage responses to MAP infection could also predict the disease state.

A main observation of the current study was the elevated numbers of CD8^+^ and TcR1-N24 γδ^+^ T cells observed in the intestinal tissue of subclinically infected cows. A recent study found increased numbers of WC1^+^ γδ T cells in focal lesions of intestinal tissue of cows naturally infected with Johne’s disease [21], with focal lesions most often associated with subclinical MAP infection. Further, using a surgically isolated bovine ileal segment model researchers found a significant increase in both CD8^+^ and γδ TCR^+^ cells in the lamina propria following infection with MAP for 9 months [22]. This model would simulate an early subclinical infection and aligns with the findings of the current study. In contrast, Koets et al. [3] observed higher numbers of TcR1-N12^+^ γδ cells in the lamina propria of clinically infected cows compared to cows in the asymptomatic stage of infection. Differences between studies may be the result of antibodies used to assess the presence of γδ cells, as binding to different epitopes would impact the results [23, 24].

No differences in the number of CD4^+^ cells between noninfected and subclinically infected cows were observed in the present study, however, the number of CD4^+^ T cells was significantly higher for both groups than was observed for clinically infected cows. In agreement with these observations, Koets et al. [3] observed significantly fewer CD4^+^ lymphocytes in the lamina propria of clinically infected cows with progressive multibacillary lesions.

The higher number of FoxP3^+^ cells observed in the mid-ileum of noninfected control cows in the present study contrasts with previous findings. Studies in red deer [25] and in cows [4, 5] have shown an increase in FoxP3 in subclinically infected animals, compared to both clinically infected and noninfected control animals. In a study by de Almeida et al. [4], ileal tissue from subclinically infected cows demonstrated an increased abundance of FoxP3 mRNA and FoxP3^+^ IHC-stained lymphocytes. However, a study by Roussey et al. [5] found a significant reduction in FoxP3^+^ cells and relative FoxP3 mRNA abundance with an increase in lesion score (indicative of disease severity) in JD infected cows. In order to explain this difference to previous work, we compared lesion score and type data collected from a previous study [17] with our FoxP3 data (Additional file 1). In our cohort of cows, we observed a total of 8/20 infected cows (7 clinically infected and 1 subclinically infected) with granulomatous lesions. Of the 7 clinically infected cows, 3 had multifocal and 4 had diffuse lesions. The subclinically infected cow had a single diffuse lesion [17]. It is likely that the low numbers of lesions observed in the current study, particularly in subclinically infected cows, may explain this contrast in FoxP3 findings with previous studies. We observed an increase in the number of FoxP3^+^ cells from multifocal to diffuse lesions and from low to higher granulomatous inflammation score in infected cows, however, cows without lesions or an inflammation score of 0 had overwhelmingly higher numbers of FoxP3^+^ cells (Additional file 1).

Clinically infected cows demonstrated significantly higher numbers of CXCR3^+^ and CCR9^+^ cells, and to the authors knowledge, this is the first study to quantify the dynamics of these particular lamina propria T cell subsets in cows naturally infected with MAP. These findings are consistent with previous studies in inflammatory bowel disease (IBD) and Crohn’s disease (CD). CD patients show increased CXCR3 expression on mononuclear cells in the inflamed mucosa [26] and IBD patients that test positive for MAP-specific IgG_1_ and IgG_2_ antibodies also show increased CXCR3 ligands in serum [27]. Further, mice challenged with live MAP showed an increase in the number of CXCR3^+^ T cells in the lamina propria, compared to a heat-killed MAP infection group and control group [27]. In a study by Gossner et al. [28], TruSeq analysis of ileocecal lymph nodes of MAP infected sheep showed consistent expression of CXCR3 and its ligand, CXCL10, in multibacillary lesions only. In contrast, the same study by Gossner et al. [28], found decreased expression of CCR9 by 4.5 fold in multibacillary lesions of JD infected sheep, but expression remained unchanged in paucibacillary lesions.

In a previous study conducted by our research group, we were able to predict the probability of both clinical and subclinical disease states using the number of macrophages (AM3K^+^ cells) in mid-ileal tissue [17]. Having already established that the number of macrophages is an important predictor of disease state, we attempted to find significant interactions between T cell markers and the macrophage marker, AM3K, that could improve these predictions. The best model used the interaction between CXCR3 and AM3K as the predictor, demonstrating that the interaction between the numbers of Th1-type T cells and numbers of macrophages at the site of infection is a significant predictor of Johne’s disease state. The PP of disease state increased with an increase in both CXCR3 and AM3K area, however, the ability to predict both subclinical and clinical disease states was improved from our previous study by the reduction in the number of AM3K^+^ cells required to obtain 100% PP. Our previous modeling indicated that a total of 2000 μm^2^ and 3000 μm^2^ of AM3K^+^ cells are required to identify both subclinically and clinically infected cows, respectively, with 100% PP [17]. In the current study, our CXCR3 and AM3K interaction model was able to predict both subclinically and clinically infected cows with 100% PP with 1000 μm^2^ of AM3K^+^ cells. In addition, the PP of both subclinical and clinical disease states remained high (99.9%) when only 500 μm^2^ of AM3K^+^ cells had been counted. The only difference between the two models was the number of CXCR3^+^ cells, with smaller counts of CXCR3^+^ cells needed to predict subclinical disease state compared to clinical disease state. This is an important finding from a diagnostic standpoint, particularly for the prediction of subclinical disease state, as subclinical infection is, by definition, devoid of clinical signs. The CXCR3 and AM3K interaction model to predict subclinical disease state has the ability to predict subclinical disease earlier during this stage of infection, allowing earlier interventions to mitigate maintenance and spread of infection. Diagnostically, counting numbers of tissue-based cells is impractical, however, a diagnostic test that can detect both CXCR3 ligands and a monocyte-derivative of AM3K in peripheral blood, perhaps in a similar way to an Ag-specific IFN-γ assay [9], may improve and allow early detection of animals subclinically infected with MAP. Studies in African buffaloes have identified induced protein (IP)-10 (CXCL10) as a potential biomarker of *M. bovis* infection via antigen recognition in whole-blood stimulation assays and ELISA [29, 30].

In summary, the findings of the current study indicate that clinically infected cows have higher numbers of CXCR3^+^ (Th1-type) and CCR9^+^ (total small intestinal T cells) cells at the site of infection when compared to subclinically infected and noninfected control cows. Further, predictive modelling indicates that the interaction between CXCR3^+^ and AM3K^+^ cells can be used as a predictor of disease state. Of diagnostic importance is the ability to predict the subclinical disease state with lower numbers of AM3K^+^ cells, which is likely due to the interaction with CXCR3^+^ cells in inflamed mucosa. Further study should be conducted to determine the practicality of utilizing a monocyte-derivative of AM3K and CXCR3 ligands in PBMCs from cows naturally infected with MAP as a diagnostic tool. This would allow the development of an assay to detect subclinically infected animals with more confidence during the early stages of the disease.

## Supplementary Information

**Additional file 1. Relationship between FoxP3**^**+**^** cells and pathology of ileum in dairy cows.** The area of FoxP3^+^ cells (μm^2^) was compared to granuloma type (None, Multifocal, Diffuse; A) and granulomatous inflammation score (0–5; B) for noninfected control cows (green), subclinically infected cows (blue) and clinically infected cows (orange).

## Data Availability

The dataset supporting the conclusions of this article is included within the article and its additional file.
