# Supplementary figures and images for: Prediction of Johne’s disease state based on quantification of T cell markers and their interaction with macrophages in the bovine intestine
Source: Vet Res. 2021 Apr 13;52:55. doi: 10.1186/s13567-021-00925-x (PMC8042692; doi:10.1186/s13567-021-00925-x)

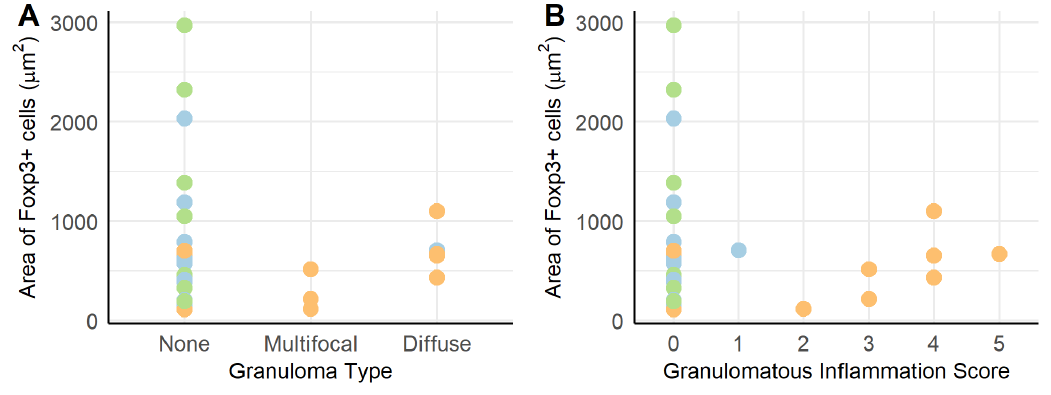

Supplement: Supplementary file 1 — Additional file 1. Relationship between FoxP3+ cells and pathology of ileum in dairy cows. The area of FoxP3+ cells (μm2) was compared to granuloma type (None, Multifocal, Diffuse; A) and granulomatous inflammation score (0–5; B) for noninfected control cows (green), subclinically infected cows (blue) and clinically infected cows (orange). [file 13567_2021_925_MOESM1_ESM.tif]
